# Evaluation of Anti-Inflammatory Effects of Celery Leaf and Stem Extracts in LPS-Induced RAW 264.7 Cells Using Nitric Oxide Assay and LC-MS Based Metabolomics

**DOI:** 10.3390/cimb43030131

**Published:** 2021-11-05

**Authors:** Hazel Lau, Nengyi Ni, Hiranya Dayal, Si-Ying Lim, Yi Ren, Sam Fong-Yau Li

**Affiliations:** 1 Department of Chemistry, National University of Singapore, 3 Science Drive 3, Singapore 117543, Singapore; hazel_lau@u.nus.edu (H.L.); chmhda@nus.edu.sg (H.D.); lim.siying@u.nus.edu (S.-Y.L.); 2 Institute of Materials Research and Engineering, Agency for Science, Technology and Research (A∗STAR), 2 Fusionopolis Way, Innovis #08-03, Singapore 138634, Singapore; reny@imre.a-star.edu.sg; 3 Department of Chemical and Biomolecular Engineering, 4 Engineering Drive 4, National University of Singapore, Singapore 117585, Singapore; e0350254@u.nus.edu; 4 NUS Environmental Research Institute (NERI), #02-01 T-Lab Building (TL), 5A Engineering Drive 1, Singapore 117411, Singapore

**Keywords:** celery, plant, inflammation, LC-MS, metabolomics, RAW 264.7 cells

## Abstract

The present work demonstrated and compared the anti-inflammatory effects of celery leaf (CLE) and stem (CSE) extracts. LC-MS-based metabolomics were an effective approach to achieve the biomarker identification and pathway elucidation associated with the reduction in inflammatory responses. The celery extracts suppressed LPS-induced NO production in RAW 264.7 cells, and CLE was five times more effective than CSE. Distinct differences were revealed between the control and celery-treated samples among the 24 characteristic metabolites that were identified. In celery-treated LPS cells, reversals of intracellular (citrulline, proline, creatine) and extracellular (citrulline, lysine) metabolites revealed that the therapeutic outcomes were closely linked to arginine metabolism. Reversals of metabolites when treated with CLE (aspartate, proline) indicated targeted effects on the TCA and urea cycles, while, in the case of CSE (histidine, glucose), the glycolysis and the pentose phosphate pathways were implicated. Subsequently, apigenin and bergapten in CLE were identified as potential biomarkers mediating the anti-inflammatory response.

## 1. Introduction

Celery (*Apium graveolens* L.), a plant of the Apiaceae family, is a leaf vegetable that originated from the Mediterranean and the Middle East and is cultivated globally. The consumption of celery is increasingly popular due to its vitamin and nutrient content and is especially beneficial for the suppression of inflammation, which is associated with multiple diseases [1]. Among the various celery parts, especially the leaves and stems, differences in chemical compositions were previously noted [2], which may have implications for their anti-inflammatory properties.

Multiple studies have attested to the anti-inflammatory potential of celery and its components. Celery extracts have been shown to suppress carrageenan-induced edema in rats [3]. Of the viable parts of celery, research into the anti-inflammatory properties of leaves was most prevalent. In mice models, celery leaf extracts reduced lipopolysaccharide (LPS)-induced miR-155 expression and tumor necrosis factor α (TNF-α) levels [4], suppressed croton-oil ear edema [5] and lowered pro-inflammatory cytokine production [6]. In cells, celery leaf extracts inhibited nitric oxide (NO) production in LPS-activated RAW 264.7 macrophages [6], as well as NO production and inducible NO synthase (iNOS) expression in LPS-activated J774.A1 macrophages [5]. For celery stem extracts, the suppression of carrageenan-induced edema in rats was reported [7]. Bioactive compounds isolated from celery also provided supporting evidence for their anti-inflammatory properties. Apiuman improved the survival of mice subjected to a lethal concentration of LPS by decreasing interleukin-1β (IL-1β) and increasing anti-inflammatory cytokine interleukin-10 (IL-10) production [8]. DL-3-n-butylphthalide reduced the inflammatory response by inhibiting the expression of pro-inflammatory cytokines and downregulating the NF-κB signal pathway in rats with LPS-induced depression [9]. LPS-induced NO production was suppressed in RAW 264.7 macrophages by sedanolide and (3S)-3-hydroxy-megastigma-5,8-dien-7-one [10], as well as in J774.A1 macrophages by apiin [5]. Through these studies, the direct anti-inflammatory effects associated with celery administration were demonstrated. However, while some relevant pathways were highlighted (e.g., downregulation of pro-inflammatory cytokine expression and cyclooxygenase inhibition), no work was carried out investigating the metabolomic changes associated with anti-inflammatory activity induced by celery.

A metabolomic analysis of RAW 264.7 cells can be used to identify the metabolites associated with the observed therapeutic effects, and was conducted using a wide variety of analytical techniques, including NMR [11,12], GC-MS [13], CE-TOF-MS [14] and LC-MS [15]. For the study of disease and associated metabolite profiles, LC-MS based metabolomics has been widely used due to its high sensitivity. LC-MS-based metabolomics has recently been demonstrated to be a valuable platform for the characterization of pro- and anti-inflammatory cell phenotypes by enabling the identification of key metabolites and perturbed pathways to provide mechanistic insights into the development of therapeutics [16].

In the present study, the anti-inflammatory properties of celery stem and leaf extracts were demonstrated through the suppression of LPS-induced NO production in RAW 264.7 cells. Subsequently, LC-MS-based metabolomics approaches were applied to investigate the intracellular and extracellular metabolome of celery-treated cells to achieve the biomarker identification and pathway elucidation associated with the reduction in inflammatory responses.

## 2. Materials and Methods

### 2.1. Materials

Dulbecco’s modified Eagle’s medium (DMEM) and fetal bovine serum (FBS) were purchased from HyClone Laboratories, Inc. (Logan, UT, USA). 1% penicillin/streptomycin was obtained from GE Healthcare (Chicago, IL, USA). Dimethyl sulfoxide (DMSO), ethanol, lipopolysaccharides (LPS), nitrite assay kit, 3-(4,5-dimethylthiazol-2-yl)-2,5-diphenyltetrazolium bromide (MTT), spermidine, histidine, serine, asparagine, glucose, citrulline, glutamate, glucose-6-phosphate, methionine, itaconate, tryptophan and FMOC-glycine were purchased from Sigma-Aldrich (St. Louis, MO, USA). Lysine, arginine, aspartate, threonine, glutamine, proline, creatine and tyrosine were purchased from Alfa Aesar (Ward Hill, MA, USA). Apigenin, apigetrin, apiin, bergapten and xanthotoxin were purchased from Toronto Research Chemicals (North York, ON, Canada).

### 2.2. Preparation of Celery Extracts

Whole celeries (individually wrapped and labeled) from Australia were purchased from local supermarkets in Singapore, and the stems and leaves were washed with deionized water before freeze-drying in a Labconco FreeZone freeze-dryer (Kansas City, MO, USA). The freeze-dried samples were ground and stored in light-protected centrifuge tubes at −80 °C before extraction. For the preparation of celery stem (CSE) and leaf (CLE) extracts, stem (0.200 g)/leaf (0.100 g) powder were added to 2 mL of 80% ethanol (*v*/*v*) adjusted to pH 1, followed by sonication at 80 °C for 2 h. Subsequently, the extract was cooled in water under ambient conditions for 5 min, centrifuged (15,400 rpm) for 10 min and filtered using a 0.2 µm PTFE filter. The supernatant was dried under vacuum, stored at −80 °C and reconstituted with DMEM before cell treatment.

### 2.3. Cell Culture and Treatment

RAW 264.7 macrophages (American Type Culture Collection, Manassas, VA, USA) were grown at standard cell culture conditions (5% CO_2_, 37 °C) in 35 mm cell culture dishes (Greiner Bio-One GmbH, Frickenhausen, Germany) with 3 mL of DMEM (HyClone, Logan, UT, USA), supplemented with 10% FBS (Gibco, Waltham, MA, USA) and 1% penicillin/streptomycin (GE Healthcare, Chicago, IL, USA). As the same culture medium was used throughout the experiments, the composition of the culture medium was not expected to impact the metabolomic findings. Cells between passages 24 and 29 were used in this study.

#### 2.3.1. MTT Assay

For cellular proliferation measurement using the MTT assay, the cells were seeded in 96-well plates at a concentration of 1 × 10^5^ cells/well. After incubation for 24 h, the cell culture medium was replaced with 100 μL of complete DMEM (medium control) or 100 μL of medium containing various concentrations of celery extract (1.25, 2.5, 5 mg/mL). After another 24 h incubation period, the medium was removed from the wells and 100 μL of MTT solution (0.5 mg/mL) was added, followed by a further 3 h incubation in the dark. Subsequently, the MTT solution was removed, and 200 μL of DMSO was added to the wells. Absorbance at 570 nm was recorded by using a Synergy HT microplate reader from Bio-Tek Instruments Inc. (Winooski, VT, USA). The absorbance was used as a measurement of cell viability, normalized to cells incubated in a control medium, which was considered 100% viable.

#### 2.3.2. NO Assay

To measure anti-inflammatory activity by NO assay, RAW 264.7 cells were seeded at 1 × 10^5^ cells/well in 96-well plates and incubated for 24 h. The cells were then treated with various concentrations of CSE (1.25, 2.5, 5 mg/mL) and CLE (1.25, 2.5, 5 mg/mL) for 1 h. Subsequently, LPS (0 or 1 µg/mL) was administered to the cells, which were then incubated for 24 h. NO production in the supernatant was evaluated by nitrite measurement based on the manufacturer’s protocol. In brief, an equal volume of supernatant and Griess reagent were mixed and incubated for 10 min at ambient temperature in the dark, and the absorbance was measured at 570 nm by a Synergy HT microplate reader from Bio-Tek Instruments Inc. (Winooski, VT, USA).

### 2.4. Metabolomics Analysis

#### 2.4.1. Collection of Culture Media and Metabolite Extraction from Cells

Aliquots of culture media were collected from each well (100 µL) into pre-cooled microcentrifuge tubes. To wash the cells, cold ultrapure water (100 µL) was added to each well and mixed with the medium aliquots, which contained extracellular metabolites. Following this, metabolite extraction from cells was conducted by adding 200 µL of pre-chilled extraction solvent (50% acetonitrile with 50 ng/mL FMOC-glycine as internal standard, −20 °C) to each well, and sonicating for 20 min in an ice bath. 50% acetonitrile was selected as the extraction solvent, as it was reported to have high extraction efficiency and did not cause interconversion or metabolite losses [17], while FMOC-glycine was chosen, as it is artificial and, therefore, suitable as an internal standard for the analysis and subsequent peak area normalization, as seen in previous studies [18,19]. The cell extracts (which contained intracellular metabolites) were collected into the second set of pre-cooled microcentrifuge tubes. Subsequently, the medium aliquots and cell extracts were filtered through 0.2 µm PTFE filters for LC-MS analysis of extracellular and intracellular metabolites, respectively. Ten biological replicates were analyzed from different wells for each condition.

#### 2.4.2. LC-MS/MS Analysis

For LC-MS/MS, the samples were analyzed on a Dionex Ultimate 3000 HPLC system (Thermo Fisher Scientific, Waltham, MA, USA) coupled to a QTRAP 5500 system (AB Sciex, Framingham, MA, USA). Multiple reaction monitoring (MRM) was employed for quantitation with an accumulation time of 5 ms per ion pair. The precursor-to-product ion pair, declustering potential (DP) and collision energy (CE) for each metabolite are described in Table 1. The chromatograms were integrated using Analyst 1.5.1 (AB Sciex, Framingham, MA, USA).

Chromatographic separation on the LC-QTrap was achieved with 5 µL injections on a Kinetex^®^ 2.6 µm Polar C18 column (100 mm × 2.1 mm, 100 Å) (Phenomenex, Torrance, CA, USA) at a flow rate of 0.35 mL/min at 35 °C, and mobile phases A and B were 0.1% formic acid in water (*v*/*v*) and 0.1% formic acid in acetonitrile (*v*/*v*), respectively. The following gradient was used: 0% B for 2 min, increased to 30% B at 4 min, ramped to 55% B at 8 min, followed by another increase to 95% B at 8.5 min, held for 2 min, then dropped to 0% B over 0.1 min and held for 4.4 min.

#### 2.4.3. Statistical Analysis

Statistical analyses were performed with R version 3.6.1 [20] (RStudio, Boston, MA, USA) using public packages, and Pareto scaling was performed before multivariate data analysis. Principal component analysis (PCA) was employed to visually discriminate the samples and identify the key metabolites responsible for the variance in the data. PCA biplots were generated using the packages ‘ggplot2′ [21] and ‘ggfortify’ [22] in RStudio (Version 1.2.5019, R. RStudio, Inc., Boston, MA, USA), which facilitates visualization of the patterns between samples and includes variable loadings to indicate metabolites associated with the separation observed between samples. Additionally, preliminary experiments showed that unstimulated cells, regardless of the presence of extract were clustered together, indicating that the extract should not have influenced the metabolomics data.

## 3. Results and Discussion

### 3.1. Effects of Celery Leaf and Stem Extracts on NO Production in RAW 264.7 Cells

**Figure 1:**
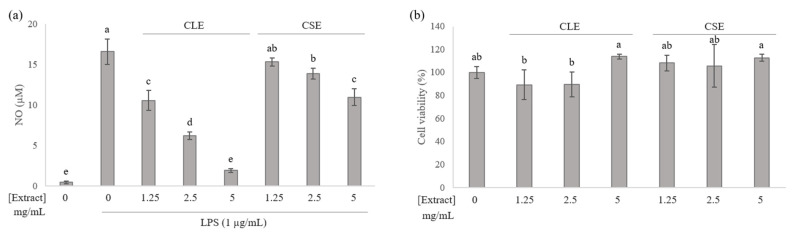
Effect of celery extracts in RAW 264.7 cells on: (**a**) nitric oxide production; (**b**) cell viability. Mean values with different letters were significantly different (*p* < 0.05).

In the present work, the anti-inflammatory activities of celery leaf and stem extracts were evaluated by measuring the suppression of NO production in LPS-induced RAW 264.7 cells. Cells were pre-treated with CLE (1.25, 2.5, 5 mg/mL) and CSE (1.25, 2.5, 5 mg/mL) for 1 h, stimulated with 1 μg/mL LPS and incubated for 24 h before analysis, while cells without any extract were considered as control groups. The addition of LPS significantly increased the NO production of cells, and the treated cells suppressed NO production in a dose-dependent manner (Figure 1a). An 8-fold and 1.5-fold suppression of NO production in the presence of CLE and CSE, respectively, was shown, with CLE demonstrating the stronger anti-inflammatory effect. Cell viability was measured using the MTT assay and appeared unaffected (>90% cell viability) in the presence of celery extracts at the tested concentrations (Figure 1b), confirming that the reduction in NO production was not due to cell death from cytotoxicity.

While the anti-inflammatory potential of celery extracts has been reported previously, such as the suppression of NO production in LPS-activated macrophages by celery leaf extracts [5,6] and the reduction in carrageenan-induced edema in rats by celery stem extracts [7], there has been no direct comparison of their anti-inflammatory activities or further work to elucidate their differences in affected metabolic outcomes.

### 3.2. Multivariate Statistical Analysis

#### 3.2.1. Intracellular Metabolites

**Figure 2:**
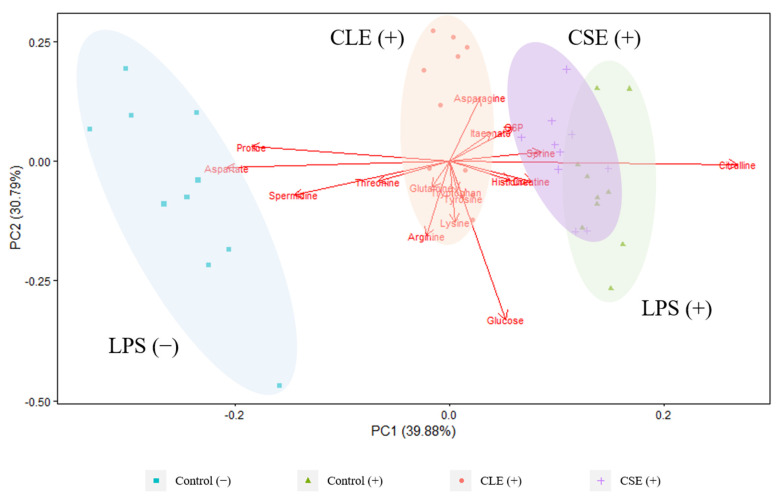
PCA biplot derived from LC-MS-targeted analysis of cell extracts from different treatment groups. (+) and (−) refer to LPS-stimulated and unstimulated cells, respectively. Red arrows represent metabolite loading vectors, and their distance from the origin indicates the extent of their influence on the PCs.

The PCA biplot derived from LC-MS targeted analysis of cell extracts showed that treatment groups representing intracellular metabolite profiles were well separated along the PC 1 axis (39.88%), with marked separation between LPS-stimulated and unstimulated cells in Figure 2. While the CLE treatment group was closer to the other LPS-stimulated groups, it was notably nearer to the unstimulated cells, indicating a reversal in intracellular metabolites that were varied due to LPS. This finding is also consistent with the more effective NO suppression in the presence of CLE (Figure 1a), and explains the poorer separation between CSE-treated and LPS-stimulated cells due to the reduced NO suppression that was observed. Intracellular citrulline was found to be strongly associated with LPS-stimulation, in agreement with multiple studies [15,16], and this is reportedly due to its role as an intermediate in NO production, which is upregulated upon LPS cell activation [23].

Higher levels of proline, aspartate and spermidine were found to contribute to the separation in unstimulated cells. Decreased intracellular proline [11] and spermidine [15] have been identified in LPS-stimulated cells, and are a result of being downstream metabolites of ornithine; LPS activation causes the diversion of arginine from ornithine production to the production of NO and citrulline due to NO synthase expression [24]. Furthermore, NO also causes the inhibition of ornithine decarboxylase, which more specifically inhibits the conversion of ornithine to putrescine for the synthesis of polyamines such as spermidine [25]. The intracellular reduction in aspartate has been identified to be due to its consumption during upregulated arginine regeneration in LPS-activated cells [15].

#### 3.2.2. Extracellular Metabolites

**Figure 3:**
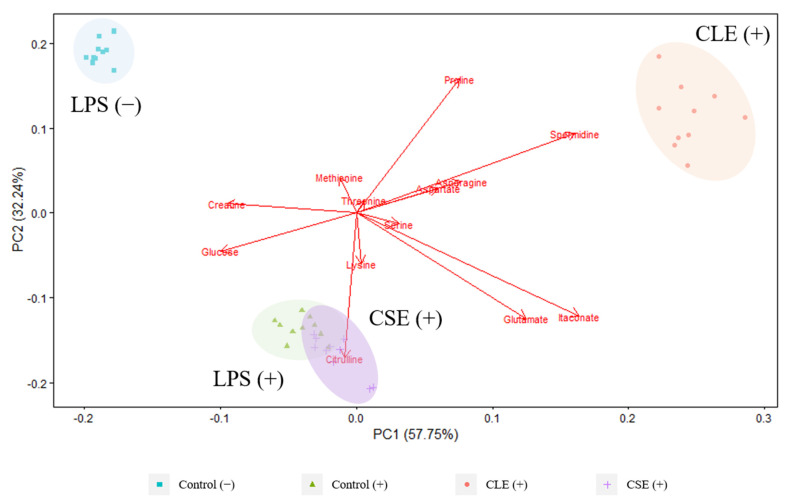
PCA biplot derived from LC-MS-targeted analysis of culture medium from different treatment groups. (+) and (−) refer to LPS-stimulated and unstimulated cells, respectively. Red arrows represent metabolite loading vectors, and their distance from the origin indicates the extent of their influence on the PCs.

Treatment groups representing extracellular metabolite profiles in the PCA biplot were mostly separated along the PC 1 axis (57.75%), and untreated LPS-stimulated and unstimulated cells were separated along the PC 2 axis (32.24%) in Figure 3. CLE-treated media was more closely associated with the unstimulated cells along the PC 2 axis, revealing clustering similarities to the intracellular metabolites and, at the same time, verifying the suppression of the LPS-induced inflammatory response by the CLE. In agreement with the trend observed with the intracellular metabolites, poor separation was also noted between LPS-stimulated and CSE-treated media. Extracellular citrulline was also strongly associated with LPS stimulation and observed in previous studies [14,23]. Lower levels of glutamate and itaconate are correlated with unstimulated cells, which are expected to be a result of the comparatively higher itaconate and glutamate production in LPS-stimulated cells due to disruptions in the tricarboxylic acid (TCA) cycle [26,27].

While there were some similarities in the separation between intracellular and extracellular metabolite profiles, it is apparent that complementary information could be obtained by using both approaches to evaluate treatment effects in cells, providing a more comprehensive overview of the changes in metabolism.

### 3.3. Heat Map Visualization

**Figure 4:**
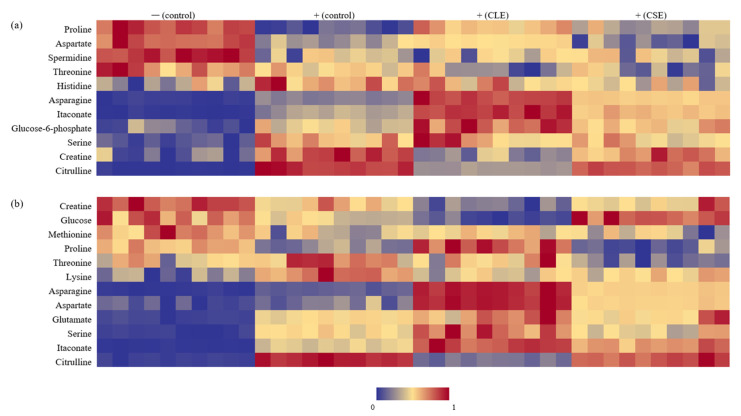
Heatmap visualization of significantly different metabolites (*p* < 0.05) derived from LC-MS targeted analysis of cell extracts and culture medium from different treatment groups. (+) and (−) refer to LPS-stimulated and unstimulated cells respectively: (**a**) intracellular metabolites; (**b**) extracellular metabolites.

**Figure 5:**
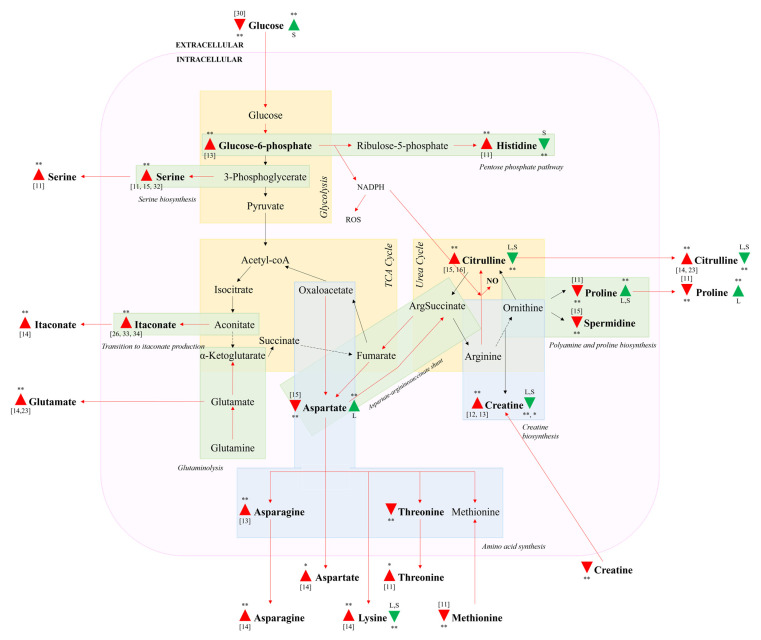
Schematic depiction of metabolic changes associated with LPS activation of RAW 264.7 macrophages and subsequent reversals with celery extracts (CLE and CSE). Compounds listed in bold are significantly differentiated (* and ** indicate *p* < 0.05 and *p* < 0.01, respectively). Red triangles indicate up/downregulation due to LPS, with reference numbers included if the finding was previously reported. Green triangles indicate up/downregulation due to celery treatment, with L/S indicating that it was caused by CLE/CSE treatment, respectively. Red arrows indicate upregulated pathways due to LPS, while black arrows indicate the pathways taken by unstimulated cells. Dotted black arrows indicate pathways suppressed by LPS.

The intracellular and extracellular metabolites responsible for the differences between LPS-stimulated and unstimulated cells (*t*-test, *p* < 0.05) are presented in Figure 4a,b. Intracellular proline, aspartate, spermidine, and threonine levels were decreased with LPS stimulation, while citrulline, creatine, serine, glucose-6-phosphate, itaconate, asparagine and histidine levels were elevated. Conversely, extracellular creatine, glucose, methionine and proline levels were lower in LPS-stimulated media, while citrulline, itaconate, serine, glutamate, aspartate, lysine and threonine were present at higher concentrations. Most of these findings have been reported previously, with the respective references for individual metabolites listed in Figure 5. It was noted that celery treatment enabled the reversal of some metabolite changes induced by LPS. Intracellular citrulline, proline and creatine were reversed by both CLE and CSE treatments, while aspartate and histidine were individually reversed by CLE and CSE treatments, respectively. Extracellular citrulline and lysine were reversed by both CLE and CSE treatments, while proline and glucose were individually reversed by CLE and CSE treatments, respectively.

In both the intracellular and extracellular samples, the reversal of citrulline was the most significant. This was anticipated, as citrulline is the most direct indicator of NO production among the analyzed metabolites [23], and the corresponding increase, associated with LPS activation as well as reduction due to celery treatment, is most closely correlated with the NO assay results (Figure 1a) and supports the anti-inflammatory activities of CLE and CSE. Reversals of intracellular proline and creatine were the most prominent, and are most likely due to their proximity to NO production in the metabolism pathways. Proline production is downregulated upon LPS activation due to the diversion of arginine for NO production [24]. While intracellular creatine is expected to be similarly reduced with the lack of available substrate (arginine) due to NO production [24], an increase was detected, which has been previously reported by two authors [12,13].

Reversals of aspartate and proline by CLE treatment specifically indicate changes that are potentially related to the TCA cycle and urea cycle—increased levels of aspartate, which can be produced by oxaloacetate and fumarate from the TCA cycle, is known to be involved in arginine regeneration via the aspartate–arginoinosuccinate shunt [26] and connects to the urea cycle upon LPS activation. In LPS-activated cells, proline production is downregulated due to arginine diversion for NO production in the urea cycle [24].

Reversals of glucose and histidine by CSE treatment specifically indicate changes that are potentially related to glycolysis and the pentose phosphate pathway (PPP)—increased glucose uptake from the media is mediated by the overexpression of glucose transporter 1 to generate energy for activated cells [28], and the corresponding increase in intracellular histidine indicates that the PPP was upregulated, which plays a role in supporting the synthesis of nucleotides, fatty acids and NADPH [29]. In particular, NADPH participates in the production of NO from arginine [24], highlighting the interdependence of the metabolic pathways.

### 3.4. Impacted Metabolic Pathways

The metabolic pathways impacted by LPS stimulation and the subsequent changes induced by celery treatment include glycolysis, the TCA cycle, and the urea cycle, as depicted in Figure 5. In LPS-activated cells, increased glucose uptake from the culture medium supports energy production for activated cells via glycolysis [30], and translates to an increase in glucose-6-phosphate. Stimulated levels of glucose-6-phosphate and histidine indicate upregulation of the PPP, and while nucleotides and proteins are also produced, increased NADPH production stimulates pro-oxidative enzymes (e.g., iNOS and NADPH oxidase), resulting in enhanced levels of reactive oxygen and nitrogen species, which induce proinflammatory gene expression [31]. Higher levels of serine were also observed due to its role in producing glycine for glutathione synthesis to induce IL-1β expression to support the inflammatory response [32]. Metabolite reversals in glucose and histidine indicate that CSE treatment targets glycolysis and the PPP, in contrast with CLE, which affects more downstream pathways such as the TCA and urea cycles.

Disruption of the TCA cycle in LPS-stimulated cells occurs with the diversion of aconitate away to itaconate production [26]. Itaconate acts via activation of nuclear factor erythroid 2-related factor 2 (Nrf2) via Kelch ECH-associating protein 1 (Keap1) alkylation to limit inflammation. This increases the expression of downstream genes with antioxidant and anti-inflammatory capacities [33], demonstrating its role in the regulation of cell inflammation. Enhanced intracellular and extracellular levels of itaconate were previously reported [14,26,33,34]. Due to the diversion of aconitate, glutaminolysis is required to replenish α-ketoglutarate to maintain the TCA cycle [27], potentially accounting for the enhanced levels of glutamate consistent with findings from two authors [14,23]. Amino acid synthesis from oxaloacetate, which appeared to be upregulated with elevated extracellular levels of asparagine, aspartate, lysine and threonine, was also previously reported [11,14]. Methionine is also produced from the same pathway, but showed a decrease in contrast with the other four amino acids, which may be explained by its additional role in S-adenosylmethionine production to facilitate the epigenetic reprogramming of histone methylation [35].

Subsequently, the TCA and urea cycles are connected by the aspartate–arginosuccinate shunt, leading to the decrease in intracellular aspartate, which functions as a substrate [15]. Most prominently, LPS activation resulted in the largest increase in both intracellular and extracellular citrulline, which was facilitated by the diversion of arginine from ornithine production due to NO synthase expression [24]. Conversely, due to the suppressed ornithine production, proline and spermidine levels were reduced. In particular, the NO-produced inhibits ornithine decarboxylase, further suppressing spermidine production [25]. Similar to itaconate, spermidine appears to regulate cell inflammation, as it inhibits the production of pro-inflammatory mediators and cytokines [36], with its anti-inflammatory properties attributed to mitochondrial superoxide-dependent 5′ adenosine monophosphate-activated protein kinase (AMPK) activation, hypoxia-inducible factor 1-α (Hif-1α) stabilization and autophagy induction [37].

### 3.5. Celery Leaf Metabolites Present in Treated Cells and Culture Medium

The anti-inflammatory activities of celery flavonoids apigenin, apigetrin and apiin, as well as coumarins bergapten and xanthotoxin, have been previously demonstrated via the suppression of LPS-induced NO production in RAW 264.7 cells [5,6,38,39]. As such, monitoring these metabolites and their uptake by cells upon LPS stimulation provides an indication of their relevance to the suppression of the detected NO production.

**Figure 6:**
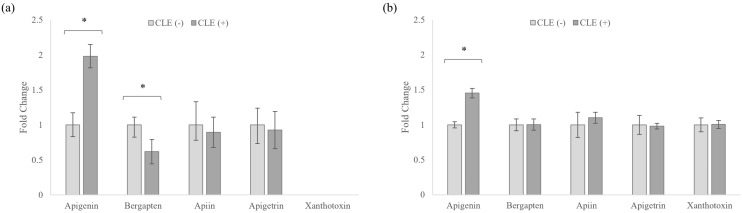
Fold change intensity variations in celery leaf bioactive metabolites in (**a**) cells; (**b**) culture medium. (+) and (−) refer to LPS-stimulated and unstimulated samples respectively. * indicates *p* < 0.05.

As these metabolites were not detected in CSE-treated cells, the discussion is focused on CLE-treated cells. From Figure 6, apigenin was significantly higher in the presence of LPS in the cells and culture media, while bergapten was significantly lower in the cells but remained constant in the culture media.

Among the flavonoids, apigenin has demonstrated the strongest inhibition of NO production in LPS-stimulated RAW 264.7 cells at 10 µM [6], which may provide evidence of its role in mediating LPS suppression. It was also noted that apiin and apigetrin, which could potentially hydrolyze to apigenin, showed consistent levels in both cells and media, indicating that the increase in apigenin was not due to degradation. Furthermore, as macrophages are not expected to produce apigenin naturally, it was likely that less apigenin was metabolized by the cells during LPS-stimulation. The reduced metabolism of apigenin, however, does not invalidate it as a contributor to the reduced inflammatory response, and future work outside the scope of this paper may seek to clarify the mechanism involved. Between the coumarins, bergapten has been reported to suppress NO production in LPS-stimulated RAW264.7 cells at 1 µM [38], which is lower than its isomer xanthotoxin, which required 25 µM [39]. As such, while xanthotoxin was below the detection limit in the cells, it is expected that bergapten would be more responsible for the suppression of NO production. These results indicate that apigenin and bergapten may be potential mediators of the anti-inflammatory response in CLE.

## 4. Conclusions

In the present study, the anti-inflammatory properties of celery stem and leaf extracts were demonstrated through the suppression of LPS-induced NO production in RAW 264.7 cells and reversal of perturbed metabolites. At the same concentration (5 mg/mL), CLE was five times more effective than CSE at reducing LPS-induced NO production. In celery-treated LPS cells, the suppression of intracellular citrulline and creatine, along with an increase in intracellular proline, were observed, in addition to reduced extracellular citrulline and lysine, indicating that the therapeutic outcomes are closely linked to arginine metabolism. Differences in the metabolic outcomes induced by celery extracts were also revealed—CLE resulted in an intracellular increase in aspartate and proline, while CSE treatment reduced intracellular histidine and increased extracellular glucose, suggesting more targeted effects on the TCA and urea cycles for CLE, in contrast with glycolysis and the pentose phosphate pathway for CSE. Subsequently, apigenin and bergapten in CLE were identified as potential mediators of the anti-inflammatory response. Overall, the anti-inflammatory effects of celery leaf and stem extracts were compared, and LC-MS-based metabolomics served as an effective approach to achieve the biomarker identification and pathway elucidation associated with the reduction in inflammatory responses.

## Figures and Tables

**Table 1:** MS parameters of cell and celery metabolites for LC-MS/MS analysis.

Analyte	MW	tR (min)	Adduct	Ionisation Mode	Q1 (m/z)	Q3 (m/z)	DP (V)	CE (eV)	CXP (V)
Spermidine	145	0.55	[M + H]+	+	146	72	40	20	6
Lysine	146	0.58	[M + H]+	+	147	84	50	23	11
Histidine	155	0.61	[M + H]+	+	156	110	50	20	11
Arginine	174	0.62	[M + H]+	+	175	70	50	30	8
Serine	105	0.63	[M + H]+	+	106	60	40	15	7
Asparagine	132	0.63	[M + H]+	+	133	74	30	20	10
Aspartate	133	0.64	[M + H]+	+	134	88	40	14	9
Threonine	119	0.65	[M + H]+	+	120	74	50	14	10
Glutamine	146	0.65	[M + H]+	+	147	130	50	13	7
Glucose	180	0.66	[M + FA − H]−	−	225	179	30	8	15
Citrulline	175	0.66	[M + H]+	+	176	159	50	14	8
Glutamate	147	0.67	[M + H]+	+	148	84	50	20	10
Glucose-6-phosphate	260	0.68	[M − H]−	−	259	97	70	21	11
Proline	115	0.71	[M + H]+	+	116	70	80	20	10
Creatine	131	0.71	[M + H]+	+	132	90	50	17	10
Methionine	149	0.92	[M + H]+	+	150	133	80	12	8
Tyrosine	181	1.55	[M + H]+	+	182	136	50	19	9
Itaconate	130	2.93	[M − H]−	−	129	85	40	12	8
Tryptophan	204	4.67	[M + H]+	+	205	188	50	14	9
Apiin	564	5.70	[M + H]+	+	565	271	40	34	14
Apigetrin	432	5.80	[M + H]+	+	433	271	60	24	15
Apigenin	270	7.10	[M − H]−	−	269	117	70	45	6
Xanthotoxin	216	7.26	[M + H]+	+	217	202	60	28	12
Bergapten	216	7.70	[M + H]+	+	217	202	60	28	10
FMOC-glycine (IS)	297	7.91	C14H10+	+	179	178	100	35	9

## Data Availability

Data are contained within the article.
